# Acute (-)-Epicatechin Consumption: Effects on Local Vasodilation Following Resistance Exercise and High-Intensity Exercise Performance

**DOI:** 10.3390/sports8020022

**Published:** 2020-02-15

**Authors:** Neil A. Schwarz, Andrew P. Theodore, Brandon R. Funderburg, Andy Waldhelm, Sarah K. McKinley-Barnard, Geoffrey M. Hudson

**Affiliations:** 1 Department of Health, Kinesiology and Sport, University of South Alabama, Mobile, AL 36688, USA; apt1521@jagmail.southalabama.edu (A.P.T.); brf1102@jagmail.southalabama.edu (B.R.F.); sarahmckinley@southalabama.edu (S.K.M.-B.); ghudson@southalabama.edu (G.M.H.); 2 Department of Physical Therapy, University of South Alabama, Mobile, AL 36688, USA; awaldhelm@southalabama.edu

**Keywords:** cocoa, dark chocolate, resistance exercise, vasodilation, brachial artery, nitric oxide, CrossFit, performance, dietary supplement

## Abstract

(-)-Epicatechin is a polyphenol previously shown to enhance vascular health. The purposes of the current studies were to determine the effect of acute (-)-epicatechin supplementation on local vasodilation in conjunction with resistance exercise (study 1) and on high-intensity exercise performance (study 2). For study 1, 11 men participated in two resistance exercise sessions, where they performed three sets of barbell curls while consuming 200 mg of 98% pure (-)-epicatechin or placebo. Measurements of total serum nitrate/nitrite and brachial artery diameter were acquired at baseline (pre-supplement), 90 min after supplement consumption (post-supplement), immediately post-exercise (post-exercise), and 30 min post-exercise (30 min post-exercise). For serum nitric oxide metabolites, no significant interaction between supplement and time nor significant main effect of time was observed (*p* = 0.38 and *p* = 0.20; respectively). For brachial artery diameter, no significant interaction between supplement and time was observed (*p* = 0.24). A significant main effect of time was observed for brachial artery diameter (*p* < 0.01) with post-exercise brachial artery diameter significantly greater diameter than all other time points (all *p* < 0.01). For study 2, six women and five men completed the 15.5 CrossFit^®^ Open Workout three times. A familiarization session was performed first where the workout was performed without the consumption of a supplement. In a randomized, balanced fashion, 100 mg of 98% pure (-)-epicatechin or cellulose (placebo) was consumed two times per day for two days before testing sessions two and three. On the day of testing sessions two and three, 60 to 90 min before completing the workout, 200 mg of the assigned supplement was ingested with water. No significant difference was observed for time to complete the workout between testing sessions (*p* = 0.49). In conclusion, under the conditions of the current studies, acute (-)-epicatechin supplementation did not augment vasodilation in combination with resistance exercise, nor did it increase exercise performance in humans.

## 1. Introduction

Cocoa polyphenols are implicated in the health benefits attributed to dark chocolate and/or cocoa consumption [1]. (-)-Epicatechin has received particular attention because it has been demonstrated to increase oxidative resistance, vasodilation, and skeletal muscle function [2,3,4]. (-)-Epicatechin is present in many foods, including grapes, green tea, and apples, but it is the most abundant polyphenol present in cocoa and cocoa-derived products [4,5,6]. Schroeter et al. [2] assessed the bioactivity of nitric oxide and flow-mediated dilation in healthy adult males as a result of high-flavanol cocoa drinks and pure (-)-epicatechin extract. Both groups displayed increased flow-mediated dilation and peripheral arterial tonometry responses only two hours after ingestion. The results indicated that (-)-epicatechin is involved in mediating the vascular effects caused by ingestion of flavanol-rich cocoa [2]. Loke et al. [3] observed that acute administration of 200 mg of (-)-epicatechin resulted in augmentation of nitric oxide production in healthy men. (-)-Epicatechin has also been shown to induce vasodilation of the femoral artery in rats upon direct administration into the bloodstream [4]. In humans, chronic supplementation with 98% pure (-)-epicatechin for four weeks inhibited aerobic exercise adaptations while having no effect on anaerobic exercise adaptations in conjunction with cycling exercise training when compared with placebo [7]. However, the ability of acute (-)-epicatechin consumption to increase vascular function [3,4] as well as increase fat oxidation rates and free fatty acid mobilization [8,9] after acute ingestion suggest it may potentially function as an ergogenic aid if consumed prior to exercise.

Previous research has studied the effect of acute dark chocolate and/or cocoa consumption on cycling time trial performance [9,10,11]. No beneficial effects on performance or nitric oxide metabolites were observed. In addition, some of the effects seen with dark chocolate consumption, e.g., fatty acid mobilization, might be due to theobromine content more so than flavanol content. Although literature exists on the effects of (-)-epicatechin on vascular function, researchers have yet to explore the effects of acute (-)-epicatechin consumption on vasodilation in conjunction with resistance training in healthy adults. Prior work by McKinley-Barnard et al. [12] demonstrated combined L-citrulline and glutathione supplementation can increase markers of nitric oxide synthesis 30 min after three sets of resistance exercise using the barbell curl, suggesting dietary supplementation can alter nitric oxide synthesis to low-volume resistance exercise. Additionally, no such studies exist that examine the effect of acute isolated (-)-epicatechin consumption on non-cycling high-intensity exercise performance in humans. Thus, the purpose of the current study was to determine the effects of acute 98% pure (-)-epicatechin ingestion on (1) brachial artery dilation and markers of nitric oxide synthesis in response to resistance exercise and (2) high-intensity exercise performance. We hypothesized that acute (-)-epicatechin ingestion would augment the vasodilatory response associated with resistance exercise and increase exercise performance.

## 2. Materials and Methods

The current study utilized two separate experimental protocols. Both experimental protocols involved a double-blind, placebo-controlled, randomized-balanced, crossover design. Different participants were recruited for each experiment. Before enrollment, participants had the protocol explained to them and provided verbal and written informed consent. For both studies, participants were free from any orthopedic injury or metabolic condition that would affect their participation. All study procedures were consistent with the ethical considerations of the Declaration of Helsinki and approved by the University of South Alabama Institutional Review Board (protocol #’s 1201717 and 1203068).

### 2.1. Experiment 1—Resistance Exercise and Vasodilation

#### 2.1.1. Participants

Eleven physically active (exercise at least 2 times weekly for six months prior to the study), healthy men, recruited by University-wide e-mail announcements, completed the study. Average age, height, body mass, and body mass index (BMI) of the participants was 21.8 ± 4.2 years (range 19 to 34 years), 1.79 ± 0.67 m, 89.4 ± 22.8 kg, and 27.7 ± 5.6 kg/m^2^, respectively. Women were excluded from this experiment to avoid potentially confounding factors as a result of their menstrual cycle [13]. 

#### 2.1.2. Protocol

Participants reported to the laboratory for one entry session and two testing sessions. During the entry session, participants had the procedures of the study explained to them followed by assessment of one-repetition maximum (1-RM) on the barbell curl exercise. The mean barbell curl 1-RM was 46.3 ± 8.8 kg. For the testing sessions, participants were required to not eat or drink anything, except water, for 10 h and to avoid exercise for 24 h prior to each session. Upon arrival, participants were asked to lay supine for 10 min—after which, resting brachial artery diameter was assessed using ultrasound and then a blood sample (10 mL) obtained (pre-supplement). Participants then ingested two capsules each containing 100 mg cellulose or (-)-epicatechin (200 mg total; Vital Pharmaceuticals, Inc, Weston, FL, USA) with water, and then rested for 90 min to allow absorption. The rationale for the dosing protocol was based on the methods of Schroeter et al. [2], where an increase in flow-mediated dilation and peripheral artery tonometry was observed two h after a single dose of 1 or 2 mg of (-)-epicatechin per kilogram of body mass. Additionally, evidence from Barnett et al. [5] suggests free (-)-epicatechin and (-)-epicatechin metabolites peak in plasma between 1 and 2 h post-ingestion in healthy volunteers. After 90 min, brachial artery diameter was assessed again followed by a second blood sample (pre-exercise). The participant then performed 3 sets of barbell curls using 65% of their 1-RM with 30 s rest between sets. The first two sets were performed for 12 repetitions each and the final set was performed for as many repetitions as possible until no more repetitions could be performed. The number of repetitions completed during the 3rd set was recorded, and the same number of repetitions was completed for the 3rd set of the second testing session. Immediately after exercise brachial artery diameter was measured and a blood sample obtained (post-exercise). The participant then rested for 30 min—after which, a brachial artery diameter measurement was performed for the last time followed by obtainment of a final blood sample (30 min post-exercise). The second session was performed in the exact same manner after 5 to 10 days while consuming the opposite supplement.

#### 2.1.3. Barbell Curl 1-RM Assessment

Participants completed a 1-RM assessment of the barbell curl exercise during the entry session. Participants grasped the barbell at shoulder width using a supine grip. Participants stood with their back against a wall to prevent excessive backward lean during execution of the repetitions. To warm up, 60% of estimated 1-RM was loaded onto the barbell and the participant performed six to ten repetitions. After a two-minute rest, the load was increased to 75% of estimated 1-RM and three to five reps were performed. The load on the barbell was then increased conservatively and one repetition was performed. After each successful repetition, participants rested for two min—after which, the load was increased, and another repetition was attempted. The highest load at which the participant could perform one repetition with proper form was recorded at the 1-RM. 

#### 2.1.4. Venous Blood Sampling

Venous blood was collected from the antecubital vein into a serum separator tube using a Vacutainer apparatus and needle (Becton, Dickinson and Company, Franklin lakes, NJ, USA). Blood samples were allowed to clot at room temperature for 10 min [14] and the samples were then centrifuged (1000 g) for 15 min and serum was aliquoted into 1.5 mL microtubes. The aliquoted samples were frozen at −80 °C for future analysis. 

#### 2.1.5. Total Serum Nitrate/Nitrite Measurement

Prior to assay, serum samples were filtered using 10-kDa molecular weight cut-off centrifugal filters (Amicon Ultra-0.5 mL Centrifugal Filters, MilliporeSigma, Burlington, MA, USA) at 14,000 g for 20 min at room temperature. Before filtration, the filters were pre-rinsed with ultrapure water. Total serum nitric oxide metabolites (nitrate/nitrite) were determined [14] using a plate-based colorimetric assay at 540 nm (SpectraMax 384 Plus, SoftMax Pro Software, Molecular Devices, San Jose, CA, USA) against a standard curve according to manufacturer specifications (Cayman Chemical; Ann Arbor, MI, USA; Item # 780001). In brief, for total nitrate/nitrite measurement, samples and standards were added to a microplate followed by the addition of enzyme cofactor mixture (Cayman Chemical, Ann Arbor, MI, USA; Item # 780012) and nitrate reductase mixture (Cayman Chemical, Ann Arbor, MI, USA; Item # 780010). The plate was covered and incubated for three h at room temperature. After incubation, Griess Reagent R1 (Cayman Chemical, Ann Arbor, MI, USA; Item # 780018) was added to each well followed by the immediate addition of Griess Reagent R2 (Cayman Chemical, Ann Arbor, MI, USA; Item # 780020). After 10 min, absorbance of the wells was measured. For nitrite measurement, samples and standards were added to a microplate followed by the addition of Griess Reagent R1 (Cayman Chemical, Ann Arbor, MI, USA; Item # 780018) and Griess Reagent R2 (Cayman Chemical, Ann Arbor, MI, USA; Item # 780020). After 10 min, absorbance of the wells was measured. Only total nitrate/nitrite values were reported because serum nitrite was undetectable in all samples at all time points. The intra-assay coefficient of variation for total nitrate/nitrite was 5.08%.

#### 2.1.6. Brachial Artery Diameter Measurement

Brachial artery diameter of the right arm was assessed using B-mode ultrasound (Mindray Z6, Shenzhen Mindray Bio-Medical Electronics, Nansham, Shenzhen, China) with a 7.5 mHz linear-array transducer with the participant in a supine position. The brachial artery was imaged above the antecubital fossa in the longitudinal plane of the medial side of the arm. Once a clear image was obtained, six separate electronic caliper measurements were manually obtained across the length of the artery image [15]. Each electronic caliper was placed using the lumen-intima interfaces as the boundaries for the diameter measurement. The six electronic caliper measurements were averaged with the resulting value used as datum.

#### 2.1.7. Statistical Analyses 

Statistical analyses for brachial artery diameter and serum nitric oxide metabolites were performed using a 2 × 4 (supplement × split time) within-within repeated measures general linear model. Post-hoc comparisons were performed using Bonferroni correction. Effect size was calculated as partial eta-squared (partial *ƞ*^2^). All analyses were performed using SPSS Statistics 22.0 software (IBM, Armonk, New York, NY, USA) with an *a priori* alpha level of 0.05.

### 2.2. Experiment 2—High-Intensity Exercise Performance

#### 2.2.1. Participants

Eleven healthy participants (6 women, 5 men) were recruited from a local CrossFit^®^ facility to participate in this experiment. Average age, height, body mass, and BMI of the participants was 26.4 ± 4.8 years, 1.72 ± 0.09 m, 75.1 ± 15.6 kg, and 25.4 ± 4.0 kg/m2, respectively. Participants had to have at least six months of experience with CrossFit^®^ style exercise training and have previously performed the 15.5 CrossFit^®^ Open Workout successfully at least once to qualify for the study. 

#### 2.2.2. Protocol

Participants completed three testing sessions consisting of the 15.5 CrossFit^®^ Open Workout. Split times for the completion of each exercise for each round were recorded along with the total time to completion for the workout. No supplement was consumed prior to the first testing session as it was treated as a familiarization workout. In a randomized, balanced fashion, 100 mg of 98% pure (-)-epicatechin (Vital Pharmaceuticals Inc., Weston, FL, USA) or cellulose (placebo) was consumed twice daily for two days before testing sessions two and three. On the day of testing sessions two and three, 200 mg of the designated supplement was consumed approximately 60 to 90 min before completing the workout. Five to seven days separated each testing session. Workouts were performed at the same time of day, and pre-testing meals were requested to be kept consistent between testing sessions. Participants were asked to refrain from performance-enhancing supplements on the day of each exercise testing session. 

#### 2.2.3. 15.5 CrossFit^®^ Open Workout

The 15.5 CrossFit^®^ Open Workout consists of rowing followed by barbell thrusters completed for four rounds with the goal to finish as fast as possible [16]. Rowing was performed using a Concept 2 Model D Rower (Concept2, Inc., Morrisville, VT, USA) at a damper level of six. To perform barbell thrusters, participants started in the standing position with a barbell placed on the shoulders. Participants then performed a full front squat and, when returning to the standing the position, the barbell was pressed overhead. After, the barbell was returned to shoulder level before initiating the next repetition. Round one consisted of rowing until expenditure of 27 kilocalories followed by 27 barbell thrusters. Round two consisted of rowing for 21 kilocalories followed by 21 barbell thrusters. Rounds three and four consisted of rowing until 15 and 9 kilocalories were expended, respectively, followed by 15 and 9 barbell thrusters, respectively. Women used a 29 kg barbell, and men used a 43 kg barbell for thrusters. Each workout was supervised and timed by a certified Level 1 CrossFit^®^ instructor. Split times were recorded after finishing each exercise of each round as well as total time to completion. 

#### 2.2.4. Statistical Analyses 

Statistical analyses for time to completion were performed using one-way repeated-measures ANOVA. Analyses for split times were performed using a 3 × 8 (supplement × split time) within-within repeated measures general linear model. Effect size was calculated as partial eta-squared (partial *ƞ*^2^). All analyses were performed using SPSS Statistics 22.0 software (IBM, Armonk, New York, NY, USA) with an *a priori* alpha level of 0.05.

## 3. Results

### 3.1. Experiment 1—Resistance Exercise and Vasodilation

**Figure 1:**
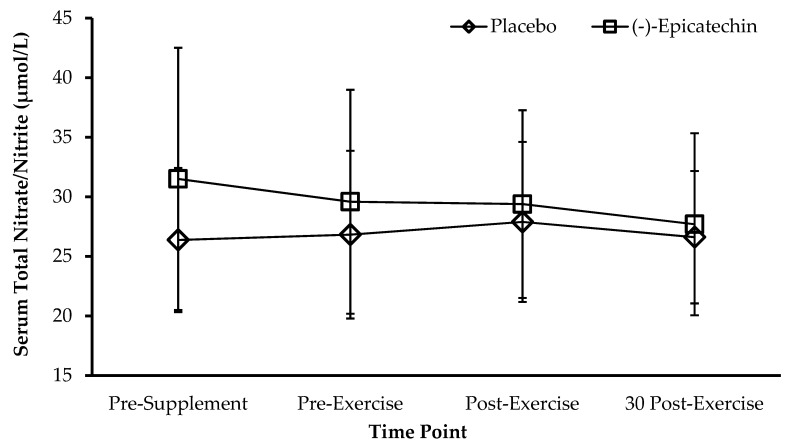
Serum total nitrate/nitrite for each condition and time point. Data presented as the mean ± standard deviation. No statistical significance between sessions.

**Figure 2:**
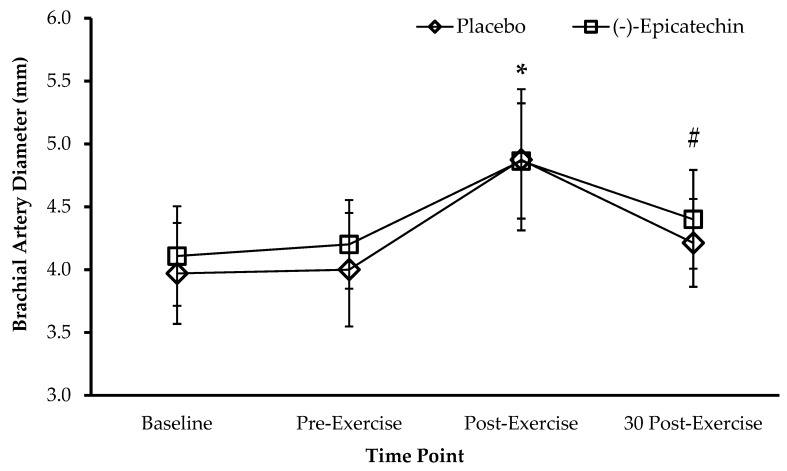
Brachial artery diameter for each condition and time point. * Brachial artery diameter at post-exercise time point is significantly greater than all other time points (all adj. *p* < 0.01). # Brachial artery diameter at 30 min post-exercise is significantly greater than pre-supplement and pre-exercise (both adj. *p* < 0.01) and significantly lower than post-exercise (adj. *p* < 0.01). Data presented as the mean ± standard deviation. No statistical significance between sessions.

For serum nitric oxide metabolites, no significant interaction between supplement and time was observed (*p* = 0.38; partial *ƞ*^2^ = 0.08; Figure 1). Furthermore, no significant main effect of time was observed (*p* = 0.20; partial *ƞ*^2^ = 0.14). For brachial artery diameter, no significant interaction between supplement and time was observed (*p* = 0.24; partial *ƞ*^2^ = 0.13; Figure 2). A significant main effect of time was observed for brachial artery diameter (*p* < 0.01; partial *ƞ*^2^ = 0.86). Post-exercise brachial artery diameter was significantly greater than brachial artery diameter than all other time points (all adj. *p* < 0.01). Brachial artery diameter at 30 min post-exercise was significantly greater than brachial artery diameter at pre-supplement and pre-exercise (both adj. *p* < 0.01). 

### 3.2. Experiment 2—High-Intensity Exercise Performance

**Figure 3:**
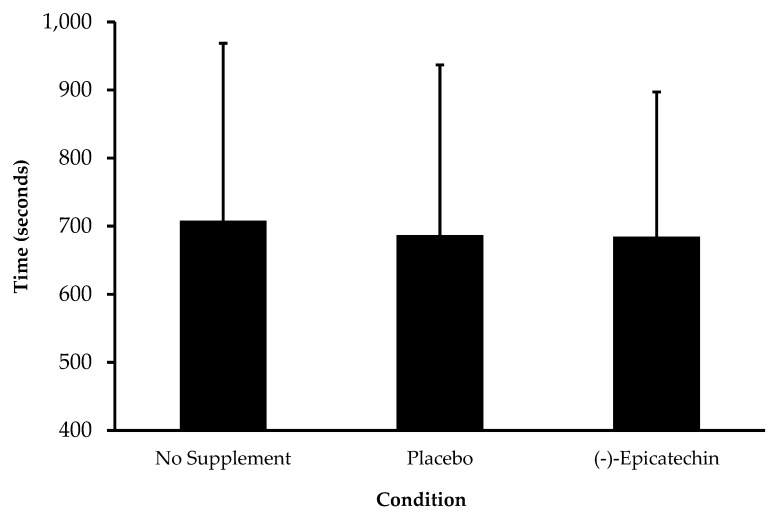
Time to completion of the 15.5 CrossFit^®^ Open Workout for each condition. Data presented as the mean ± standard deviation. No statistical significance between sessions.

**Figure 4:**
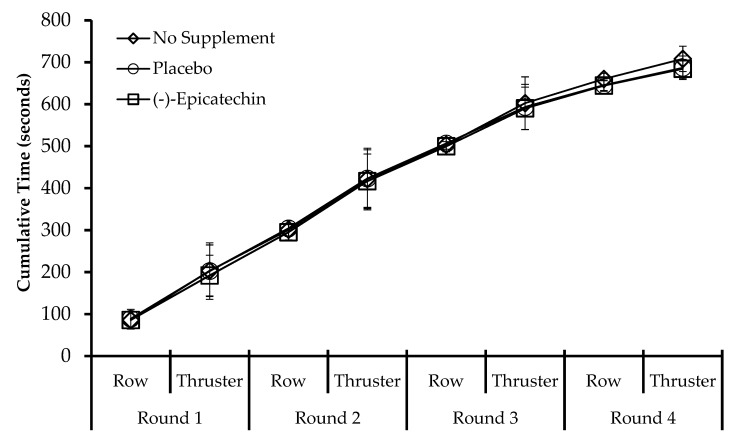
Cumulative split times for each exercise and round plotted by each session. Data presented as the mean ± standard deviation. No statistical significance between sessions.

No significant difference was observed for time to complete the workout between testing sessions (*p* = 0.49; partial *ƞ*^2^ = 0.07; Figure 3). Additionally, no significant interaction between supplement and split time was observed (*p* = 0.19; partial *ƞ*^2^ = 0.16; Figure 4). Further analyses also revealed no influence of testing session order or sex on the time to complete the workout. 

## 4. Discussion

The main findings of the current studies are that acute (-)-epicatechin supplementation did not augment vasodilation in conjunction with resistance exercise and acute (-)-epicatechin consumption did not enhance high intensity exercise performance. Brachial artery diameter significantly increased as a result of resistance exercise. Despite previous reports of increased vascular function as a result of (-)-epicatechin supplementation [2,17], the participants of this study did not demonstrate augmented brachial artery diameter with supplementation at rest or after exercise. Maximal brachial artery diameter response was almost identical between the two testing sessions; thus, it is possible that, in healthy young men, brachial artery responses to resistance exercise are robust and unable to be further enhanced by acute (-)-epicatechin supplementation. It is important to point out that the assessment of only brachial artery diameter limits the scope of study to the large vasculature structures. It is feasible that (-)-epicatechin supplementation was able to increase blood delivery to skeletal muscle through increases in capillary recruitment; however, it was not possible to evaluate this with the current study design. It is also feasible that blood flow may have been enhanced by (-)-epicatechin supplementation without observed changes in artery diameter. Furthermore, in other studies demonstrating increased flow-mediated dilation and vascular function, (-)-epicatechin was consumed in combination with other flavonoids and/or as a cocoa or dark chocolate product [18,19,20]. Hence, it may be important for (-)-epicatechin to be consumed in a different medium or in combination with other flavonoids or nutrients. 

Interestingly, even though large increases in brachial artery diameter were observed, serum nitric oxide metabolite concentration remained unchanged. A previous study with a similar resistance exercise design reported an increase in nitric oxide metabolites at similar time points as used in this study [14]. In contrast, in the current study, three sets of barbell curls with minimal rest was not sufficient to increase nitric oxide metabolites in venous blood. Additionally, serum nitrite levels in the present study were too low to be detected at any time point. In agreement with our results, acute cocoa flavanol consumption did not increase nitric oxide metabolites during a time trial in well-trained male cyclists [11] suggesting neither cocoa consumption nor pure (-)-epicatechin consumption are capable of inducing increases in nitric oxide production in combination with exercise in healthy young men. It is likely that a more pronounced exercise intervention is required to induce consistent elevation in nitric oxide metabolites in venous blood and this finding should be considered for future study protocol design. For example, a 20 min cycling protocol at 90% of the anaerobic threshold was unable to increase nitric oxide metabolites in healthy participants [21]. Alternatively, cycling for 2 h was shown to increase nitric oxide metabolites by ~16% to 18%, and this effect was greater in athletes versus non-athletes [22]. Even further, completion of a 42.195 km marathon race doubled plasma nitric oxide in healthy males between 21 and 39 years of age [23]. Thus, much longer exercise durations employing greater muscle mass may be needed to appropriately induce increases in nitric oxide formation; and, although the participants of study 1 were physically active, they may not have possessed the physical fitness level necessary to provide augmented nitric oxide formation. 

In response to exercise stress, blood flow is diverted to working skeletal muscle in order to increase oxygen and nutrient delivery and to remove waste products and byproducts of increased metabolism [24]. (-)-Epicatechin, when consumed as cocoa extract or dark chocolate, has been shown to increase flow-mediated dilation and fat oxidation after acute consumption [2,11]. Consumption of (-)-epicatechin could purportedly increase exercise performance secondary to enhanced oxygen delivery, waste removal, and fat metabolism; thus, (-)-epicatechin supplementation was evaluated as an ergogenic aid prior to high-intensity exercise. No improvement was observed for time to complete the 15.5 CrossFit^®^ Open Workout with (-)-epicatechin supplementation suggesting that, for high-intensity exercise lasting approximately 11 to 13 min, (-)-epicatechin supplementation does not offer an ergogenic benefit. This is consistent with three previous studies where dark chocolate and cocoa consumption did not increase cycling time-trial performance [9,10,11]. Thus, (-)-epicatechin does not appear to be an effective ergogenic aid in humans when consumed in isolation or as a component of cocoa. It remains undetermined if acute (-)-epicatechin supplementation can enhance exercise performance for longer duration events lasting over 30 min or more or exercise events employing different modalities. These studies contain important limitations that should be addressed. First, dietary intake of nitrates through food was not directly controlled. Participants were asked to avoid foods such as dark chocolate and green tea; however, this was not confirmed through dietary monitoring. Further, sample size for each study was relatively small and may have reduced the ability to detect meaningful differences. Importantly, a major limitation of both studies is that (-)-epicatechin nor (-)-epicatechin metabolites were measured to determine the effectiveness of the supplementation protocol on bioavailability. For study two, the lack of control for the menstrual cycle phase of female participants may have confounded the results regarding exercise performance.

## 5. Conclusions

In conclusion, acute (-)-epicatechin supplementation, ingested as a single dose, did not augment vasodilation in combination with resistance exercise in young, physically active men. Additionally, time to completion of a high-intensity exercise test was not improved as a result of acute supplementation of (-)-epicatechin, over a 3 day period, in active men and women. The potential for (-)-epicatechin to augment vascular responses to resistance exercise or exercise performance still exists. Utilization of alternative dosing strategies (e.g., higher amount, greater duration), different exercise protocols, and/or different target populations (e.g., older, diseased) may produce differing results and should be explored.
